# Methotrexate Treatment Causes Early Onset of Disease in a Mouse Model of Ross River Virus-Induced Inflammatory Disease through Increased Monocyte Production

**DOI:** 10.1371/journal.pone.0071146

**Published:** 2013-08-12

**Authors:** Adam Taylor, Kuo-Ching Sheng, Lara J. Herrero, Weiqiang Chen, Nestor E. Rulli, Suresh Mahalingam

**Affiliations:** Institute for Glycomics, Griffith University, Gold Coast Campus, Queensland, Australia; University of Georgia, United States of America

## Abstract

Part of the *Togaviridae* family, alphaviruses, including chikungunya virus (CHIKV), Sindbis virus (SINV) and Ross River virus (RRV), are able to cause significant inflammatory pathologies ranging from arthritis to encephalitis. Following symptomatic infection with arthritis-associated alphaviruses, patients often experience severe joint pain, affecting distal and small joints, which can last six months or longer. Recently, methotrexate (MTX), a disease modifying anti-rheumatic drug (DMARD), was used to treat patients experiencing chronic rheumatic symptoms following infection with CHIKV. Here, the effect of MTX on Ross River virus disease (RRVD) in mice was examined to better understand its therapeutic potential for alphaviral-induced musculoskeletal disease and to further our knowledge of the development of alphaviral pathologies. Using a mouse model, we analyzed the effect of MTX on RRVD. RRV disease pathogenesis in response to MTX treatment was determined by measuring levels of proinflammatory factors, cellular infiltrates, viral titer and histological analysis of infected tissues. RRV-infected mice receiving MTX treatment rapidly developed musculoskeletal disease, which correlated with a significant influx of inflammatory cell infiltrates into the skeletal muscle tissue. Although no difference was observed in the level of proinflammatory cytokines and chemokines, the viral load increased at early time points post infection in the serum and quadriceps of MTX treated mice, possibly contributing to disease pathogenesis. Results suggest that MTX treatment of acute RRVD in mice provides no therapeutic benefit and underline the importance of inflammatory monocytes in alphaviral induced arthritides.

## Introduction

Arthropod-borne alphaviruses are globally widespread and capable of causing significant inflammatory pathologies. Outbreaks of Old World alphaviruses, such as chikungunya virus (CHIKV), Sindbis virus (SINV) and Ross River virus (RRV), are largely associated with highly debilitating arthritic symptoms, causing significant human morbidity.

RRV is endemic to Australia and Papua New Guinea where it is responsible for increasingly frequent outbreaks of polyarthritis/arthralgia. In 2009, 4,786 cases of Ross River virus disease (RRVD) were reported in Australia [1]. In Western Australia the number of cases of RRV reported in January and February, rose from 245 in 2011 to 632 for the same period in 2012 [2]. Classically, patients infected with RRV present with a febrile illness, arthritis, malaise and a maculopapular erythematous rash, commonly affecting the limbs and trunk. While the prevalence of RRV in Papua New Guinea remains unclear due to inadequate detection, it is suggested that RRV could emerge as one of the most common infections of Oceania’s poorest people within the next decade [3]. Furthermore, the severe chronic and recurrent arthralgia experienced by infected patients, together with the epidemic nature of RRV outbreaks, makes RRVD an illness of major socioeconomic concern [4].

The immunopathological mechanisms responsible for alphaviral-induced arthropathies are diverse and still poorly understood. In patients infected with arthritogenic alphaviruses, including RRV, migration of macrophages and monocytes into the synovium suggests that these cells may play an important role in the inflammation associated with alphaviral disease [5], [6]. In vivo systems, such as the established mouse model of RRVD, also play an important role in identifying host and viral factors that mediate alphaviral-induced disease. These models highlight the importance of myeloid monocytes/macrophages in alphaviral-induced tissue inflammation. Pronounced macrophage infiltrates in mouse joint and skeletal muscle tissues were observed following infection with CHIKV and RRV [7], [8]. Additionally, systemic depletion of monocytes/macrophages substantially ameliorated joint and muscle inflammation in the mouse model of RRVD [7].

Current treatments for viral arthropathies rely on non-steroidal anti-inflammatory drugs (NSAIDs), however these often only provide partial relief [9]. A number of patients who experienced chronic rheumatic symptoms following infection with CHIKV during the 2005–2006 La Réunion outbreak were successfully treated with methotrexate (MTX) [6]. Originally developed as a chemotherapeutic, MTX has become the cornerstone of most rheumatoid arthritis (RA) treatment regimens due to its anti-inflammatory effects at low doses [10]. Despite this, details of its anti-inflammatory mechanism and its effect on acute viral induced arthritis remain unclear. In this study we examined the effect of MTX treatment on a mouse model of RRVD. RRV infected mice treated with MTX quickly developed RRVD signs when compared to mock-treated mice. Accelerated disease onset was accompanied by an increase in monocyte infiltration of skeletal muscle tissues together with a systemic increase in inflammatory monocyte cellularity. Importantly, MTX treatment of RRVD in mice provided no therapeutic benefit.

## Materials and Methods

### Virus

Stocks of the T48 strain of RRV were generated by transcription of the full-length T48 complementary DNA (cDNA) clone [11]. The transcripts were electroporated into Vero cells and supernatants collected and titrated by plaque assay as described below.

### Mice

21-day-old C57BL/6 mice were subcutaneously inoculated in the thorax (below the right forelimb) with 10^4^ plaque-forming units (PFU) of RRV diluted in phosphate buffered saline (PBS) in a 50 µl volume. Mock-infected mice were injected with diluent alone. Mice were scored for disease symptoms and weighed every 24 hours as described previously [12] and sacrificed by CO_2_ asphyxiation at experimental end points. This study was approved by the Animal Ethics Committee of Griffith University.

### Methotrexate Treatment of Mice

Mice were injected intraperitoneally (IP) with methotrexate (Sigma-Aldrich ***06563***, 0.25 mg/kg) or PBS in a 100 µl volume. Treatment was performed daily and commenced either on the day of RRV infection or on day 6 post infection.

### Viral Titer Assay

Mice were sacrificed at days 1, 3, 5 and 10 post infection with the quadriceps tissue, ankle joint and serum collected and assayed for viral titer using plaque assay. Tissue samples were homogenized in 1 mL of PBS and 10-fold serial dilutions of homogenate and sera were added in triplicate to Vero cells. Virus was allowed to incubate for 1 h at 37°C in a 5% CO_2_ incubator before virus was removed and the cells overlaid with OPTI-MEM (Invitrogen, Melbourne, Victoria, Australia) containing 3% FCS and 1% agarose (Sigma Aldrich, Sydney, Australia) and incubated for 48 h in a 5% CO_2_ incubator. Cells were fixed in 1% formalin and virus plaques were made visible by staining with 0.1% crystal violet. Results were expressed as plaque forming units per millilitre (PFU/mL) or PFU per gram of tissue.

### Quantitative RT-PCR

RNA was extracted from tissues using TRIzol (Invitrogen, Melbourne, Victoria, Australia) according to the manufacturer’s instructions. 1 µg of RNA was reverse transcribed using random primers and reverse transcriptase (Sigma Aldrich, Sydney, Australia) according to the manufacturer’s instructions. Quantitative PCR was performed with 50 ng of template cDNA, QuantiTect Primer Assay kits (Qiagen, Hilden, Germany) and SYBR® Green Real-time PCR reagent in a CFX96 Touch™ Real-Time PCR System using a standard three-step melt program (95°C for 15 s, 55°C for 30 s and 72°C for 30 s). Data were normalized to the housekeeping gene HPRT1 and the fold change in messenger RNA (mRNA) expression relative to mock-infected PBS treated samples for each gene was calculated using the ΔΔCt method. Briefly, ΔΔCt = ΔCt (RRV-infected) – ΔCt (Mock-infected) where ΔCt = Ct (gene of interest) – Ct (housekeeping gene - HPRT). The fold change for each gene is calculated as 2^−ΔΔCt^.

### Flow Cytometry

Quadricep muscles were collected from mice and weighed. They were then minced and treated with 200 µl of RPMI media containing 1 mg/ml DNase I (Sigma Aldrich) and 3 mg/ml Collagenase IV (Worthington, Lakewood, NJ) at 37°C for 1.5 h. The tissue mass was mixed with 5 mL RPMI, vigorously pipetted for 10 sec, and passed through a 40 µm cell strainer. Cells were washed, pelleted and treated with 1× RBC lysis buffer (BD Biosciences) for 5 min, and counted. Similar to quadriceps, spleens were minced, strained and treated with lysis buffer for splenocyte analysis. To determine percentages and numbers of specific leukocyte populations, cells were first treated with Fc Block (2.4G2; BD) for 5 min at 4°C and labeled with fluorochrome-conjugated anti-mouse antibodies, including anti-CD3-FITC (145-2C11, BD), anti-CD19-APC (MB19-1, eBioscience), anti-CD11b-PE (M1/70, BD), anti-Gr1-APC (RB6-8C5, eBioscience), anti-Ly6C-APC (HK 1.4, eBioscience), anti-pan-NK/NKT antigen-PE (U5A2-13, BD) and anti-CD45-PE-Cy5 (30-F11, eBioscience), in various combinations at 4°C for 30 min. To detect inflammatory monocytes/macrophages, we found that CD11b was a more distinguishable marker than F4/80 in quadriceps. Cells were resuspended in 500 µL PBS containing 2% FCS and 1 µg/mL propidium iodine (PI), and analysed by the CyAn ADP flow cytometer (Beckman Coutler) with Kaluza software.

### Histological Analysis

Mice were sacrificed at day 6 and day 10 post infection with the left quadriceps collected and fixed in 4% paraformaldehyde (PFA) for 3 days. Paraffin-embedded, hematoxylin and eosin (H&E)-stained longitudinal sections were prepared by The Queensland Institute for Medical Research (QIMR) and The University of Queensland (UQ) Faculty of Health HistoTechnology Facility. Slides were examined under an Olympus BX60 microscope.

### Statistics

Student’s unpaired *t*-tests were used to analyze differences in flow cytometry and virus titer analyses. To examine more than two groups of flow cytometry data one-way ANOVA analyses were performed. One-way ANOVA analyses were also performed to analyze qRT-PCR and histological cell counts. Man-Whitney U tests were used to analyze mouse clinical disease score and two-way ANOVA with Bonferroni post-tests were used to analyze mouse weight. A *p* value <0.05 was considered to be significant.

## Results

### MTX Treatment Accelerates RRVD Onset in Mice

**Figure 1:**
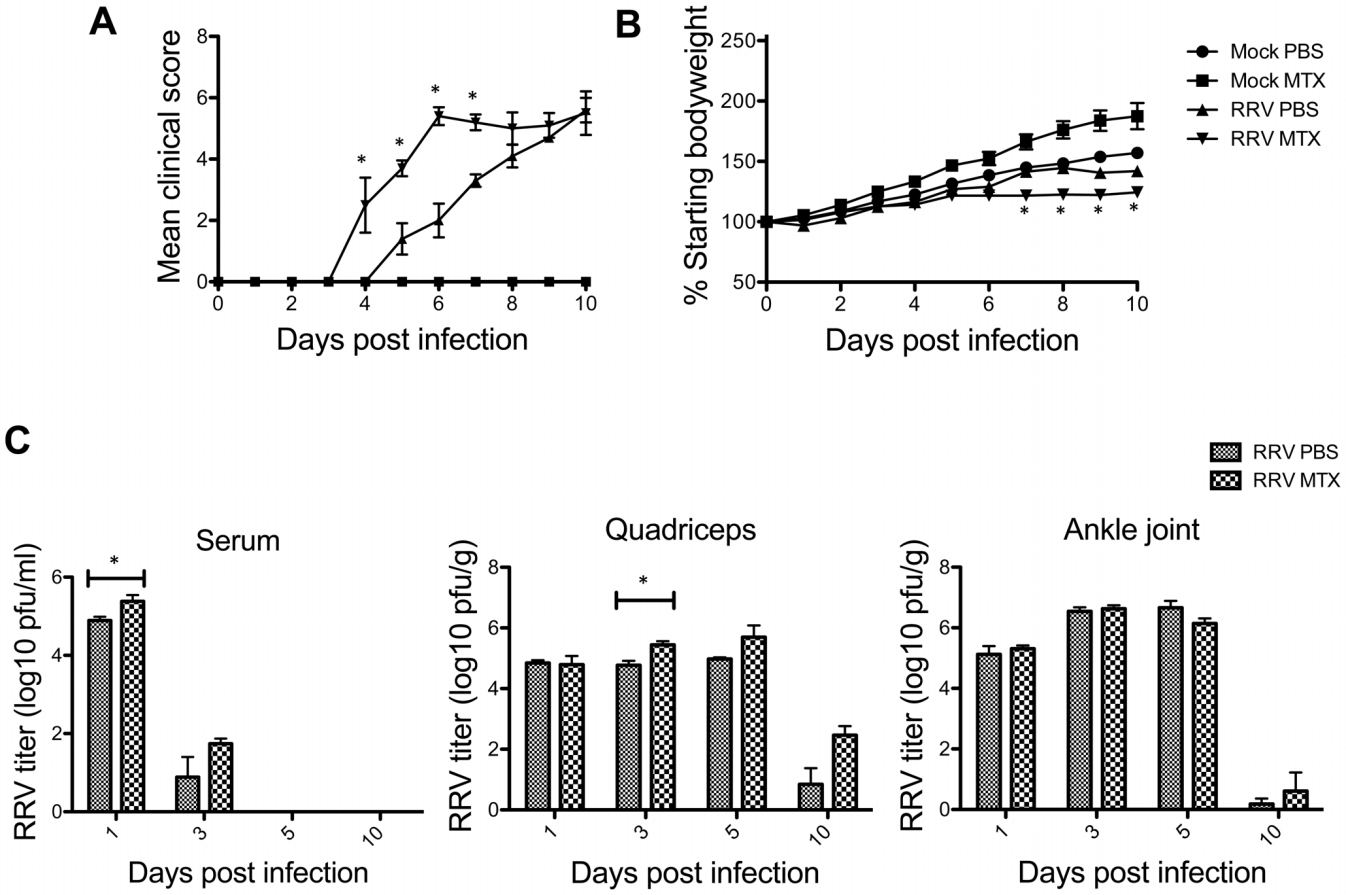
MTX treatment leads to early onset of RRVD in mice. Mice were subcutaneously injected with 10^4^ PFU of RRV and mock inoculated mice injected with PBS alone. Mice were treated with MTX or PBS intraperitoneally from the day of infection. (A) Mice were scored daily (0–8 scale) for the development of hind limb dysfunction and disease as described previously [12]. Values are the mean ± SEM for 5 mice per group. * = *P* < 0.05 versus RRV PBS mice. (B) Mice were monitored daily for changes in weight. Values are the mean ± SEM for 5 mice per group. * = *P* < 0.05 versus RRV PBS mice. (C) At day 1, 3, 5 and 10 post infection, serum, quadriceps and ankle joints from infected mice were collected and the amount of infectious virus present quantified by plaque assay on Vero cells. Values are the mean ± SEM for 5 mice per group. * = *P* < 0.05.

**Figure 2:**
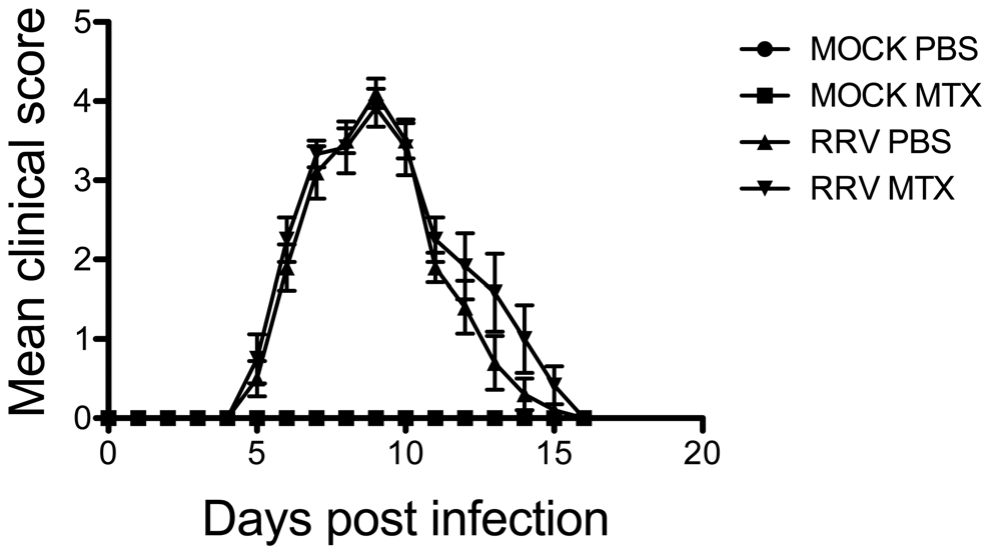
MTX has no therapeutic effect on RRVD in mice. Mice received intraperitoneal injections of MTX or PBS from day 6 post infection. Mice were scored daily (0–8 scale) for the development of hind limb dysfunction and disease as described previously [12]. Values are the mean ± SEM for 5 mice per group.

To analyze the effect of MTX on RRVD, mice were infected with 10^4^ PFU of RRV and received daily IP injections of MTX or PBS control commencing on the day of infection. Mice were monitored daily for the development of disease signs and weight gain. RRVD symptoms in infected mice typically range from low scoring mild signs such as increased lethargy and delicate walking to complete loss of hind limb function at peak disease. Mock-treated RRV-infected mice began displaying signs of disease at day 5 post infection, with peak disease observed at day 10. Interestingly, infected mice receiving MTX treatment showed a rapid onset of severe clinical signs, with complete loss of hind limb function by day 6 post infection (Figure 1A). Furthermore, MTX-treated infected mice showed a significant decrease in bodyweight between day ***7*** and 10 post infection when compared to mock-treated infected control mice (Figure 1B). The therapeutic effect of MTX on RRVD in mice was analyzed by delaying treatment until the onset of clear disease signs (day 6 post infection). The clinical score of infected MTX-treated mice mirrored that of mock-treated mice following delayed treatment, suggesting MTX treatment of RRVD in mice has no therapeutic benefit (Figure 2).

**Figure 3:**
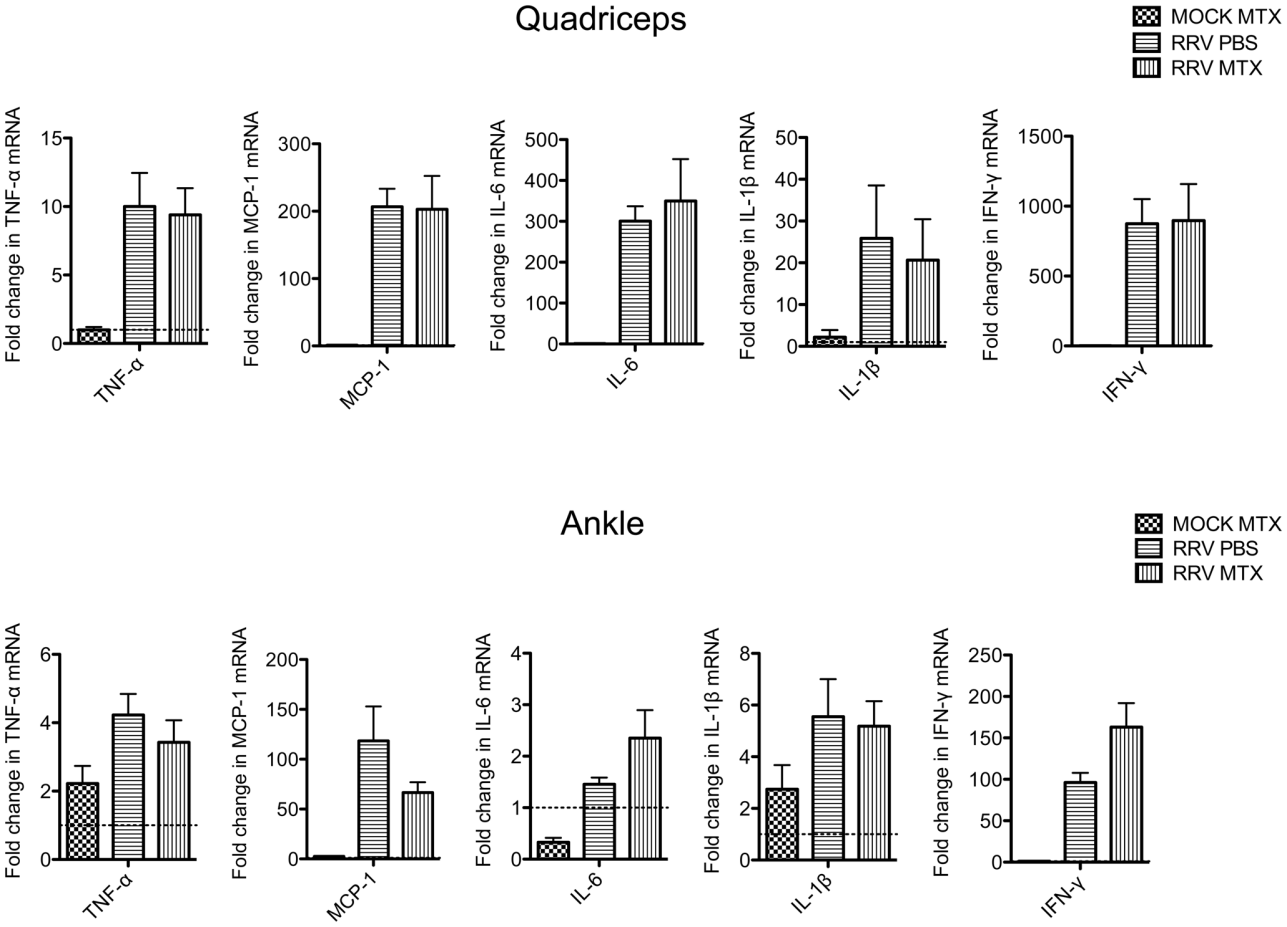
MTX has no effect on the expression of proinflammatory factors in quadriceps and ankle tissues at day 6 post infection. Mice were subcutaneously injected with 10^4^ PFU of RRV and mock inoculated mice injected with PBS alone. Mice were treated with MTX or PBS intraperitoneally from the day of infection. Total RNA was extracted from quadriceps and ankle tissues on day 6 post infection and fold change in expression of TNF-α (tumor necrosis factor-alpha), IFN-γ (interferon-gamma), MCP-1 (monocyte chemoattractant protein 1), IL-1β (interleukin-1 beta) and IL6 (interleukin 6) analysed by qRT-PCR. Values are the mean ± SEM for 5 mice per group.

To further investigate the early onset of RRVD in mice receiving MTX treatment from the day of infection, RRV titer was measured in the ankle joints, skeletal muscle and serum of MTX and mock treated mice. Differences were observed in serum and quadriceps viral titers between mock and MTX-treated mice at early time points. MTX-treated mice showed a significant increase in viral titer at day 1 and day 3 post infection in sera and quadriceps respectively which may contribute to the rapid development of severe disease in RRV-infected mice receiving MTX (Figure 1C). We also examined the levels of proinflammatory factors in quadriceps and ankle tissues at day 6 post infection. To examine whether the expression of proinflammatory factors was altered in mice receiving MTX treatment from day 0, mRNA levels were measured by qRT-PCR. No significant difference in TNF-α, IFN-γ, MCP-1, IL-1β or IL-6 mRNA levels was observed at day 6 post infection in muscle or ankle tissues in infected mice receiving mock or MTX treatment (Figure 3).

### MTX Treatment Enhances Immune Infiltration Early in RRV Infection, in Parallel to Increased Generation of Systemic Inflammatory Monocytes

**Figure 4:**
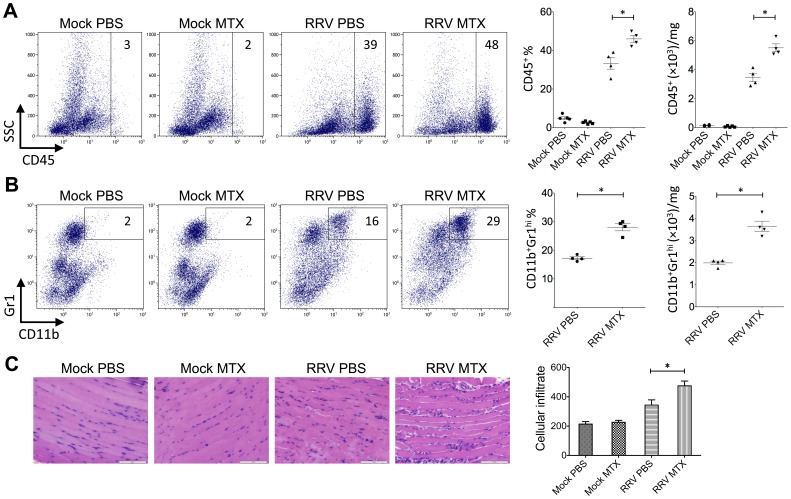
MTX treatment of RRVD in mice accelerates skeletal muscle infiltration of inflammatory monocytes at day 6 post infection. Mice were injected subcutaneously with 10^4^ PFU of RRV and mock inoculated mice injected with PBS alone. Mice received intraperitoneal injections of MTX or PBS from the day of infection. The total number and percentage of (A) CD45^+^ leukocyte and (B) CD11b^+^ monocyte cells was determined by flow cytometry of whole quadriceps on day 6 post infection. Values are the mean ± SEM for 5 mice per group. * = *P* < 0.05. (C) Quadriceps were collected, fixed in 4% paraformaldehyde, sectioned and stained with hematoxylin and eosin. Images and infiltrate quantification are representative of 6 mice per group. Magnification is 200X and the white bar represents 100 µm. * = *P* < 0.05.

**Figure 5:**
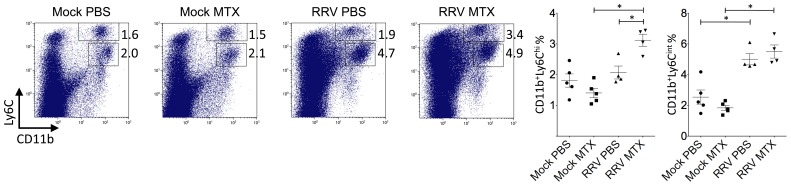
MTX treatment of RRVD in mice increased the percentage of systemic inflammatory monocytes at day 6. Mice were infected with 10^4^ PFU RRV and treated with MTX as described. Total numbers and percentages of splenic CD11b^hi^Ly6C^hi^ monocytes and CD11b^+^Ly6C^int^ neutrophils were determined by flow cytometry at day 6 post infection. Values are the mean ± SEM for 4–5 mice per group. * = *P* < 0.05.

To further investigate why MTX treatment accelerated the RRVD onset and sustained peak disease, cell infiltration in infected quadriceps muscles was determined by flow cytometry. We have previously established the critical role of CD11b^+^F4/80^+^Gr1^+^ inflammatory monocytes/macrophages in the severity of RRV disease [12]. Based on the disease kinetics (Figure 1A), mice were sacrificed at day 6 and 10 post infection representing the biggest difference in clinical score and similar peak disease between MTX and mock-treated RRV-infected mice, respectively. Cellular infiltrates in the skeletal muscles were analyzed. As shown in Figure 4A, all RRV-infected mice exhibited substantial infiltration of CD45^+^ leukocytes in quadriceps, in comparison to the control groups. However, MTX-treated mice showed considerably more leukocyte infiltration in infected quadriceps than that in mock-treated mice at day 6, both in percentages and numbers. Subsequently, we identified a significant increase of myeloid CD11b^+^Gr1^+^ inflammatory monocyte infiltration within the tissue of MTX-treated mice (Figure 4B), while other minor infiltrates including T, B and natural killer (NK) cells remained comparable (data not shown). In line with the enhanced leukocyte infiltration observed by flow cytometry, histological analysis revealed an increase of cellular infiltrates, with enhanced tissue damage, in infected quadriceps of MTX-treated mice infected with RRV (Figure 4C). These results suggest that MTX enhances myeloid monocyte infiltration during RRV infection. Because the spleen represents a bona fide reservoir for inflammatory monocytes in circulation, which are recruited to the local tissue in response to inflammation [13], we investigated whether MTX altered the myeloid immune compartment at the systemic level. As shown in Figure 5, in the context of RRV infection, MTX treatment facilitated the cellularity of systemic CD11b^+^Ly6C^hi^ inflammatory monocytes, but not CD11b^+^Ly6C^int^ neutrophils in the spleen, suggesting a role for MTX in specific enhancement of myeloid inflammatory monocyte generation during RRV infection, which could subsequently lead to disease acceleration.

### MTX Treatment Sustains the Level of CD11b^+^Gr1^hi^ Monocyte Infiltration and Converges with that of Control Mice at Day 10 of RRV Disease

**Figure 6:**
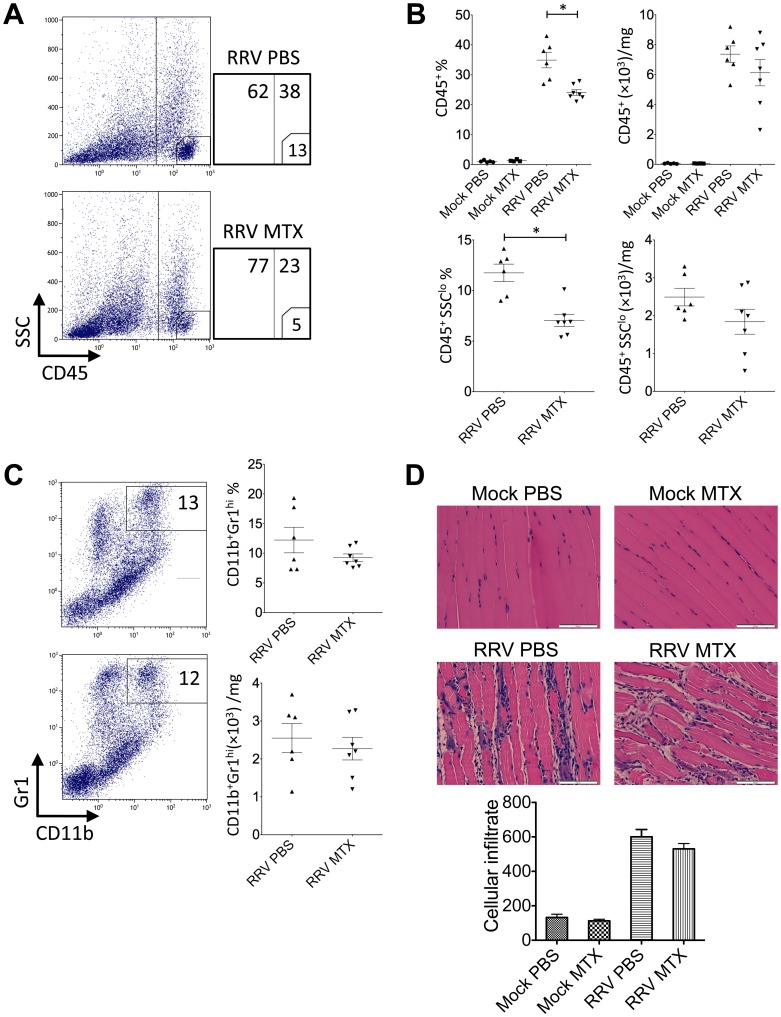
MTX treatment has no effect on the number of inflammatory cell infiltrates at day 10 post infection. Mice were injected subcutaneously with 10^4^ PFU of RRV and mock inoculated mice injected with PBS alone. Mice received intraperitoneal injections of MTX or PBS from the day of infection. The total number and percentage of (A and B) CD45^+^ leukocyte and (C) CD11b^+^ monocyte cells was determined by flow cytometry of whole quadriceps on day 10 post infection. Values are the mean ± SEM for 5 mice per group. * = *P* < 0.05. (D) Quadriceps were collected, fixed in 4% paraformaldehyde, sectioned and stained with hematoxylin and eosin. Images and infiltrate quantification are representative of 6 mice per group. Magnification is 200X and the white bar represents 100 µm.

**Figure 7:**
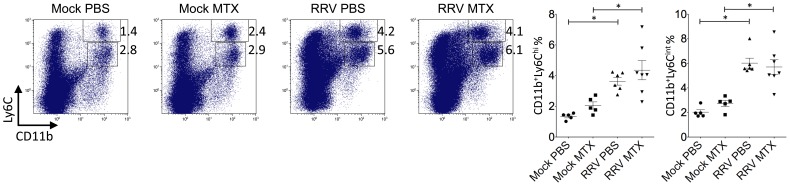
MTX treatment of RRVD in mice has no effect on the percentage of systemic inflammatory monocytes at day 10. Mice were infected with RRV and treated with MTX as described. Total numbers and percentages of splenic CD11b^hi^Ly6C^hi^ monocytes and CD11b^+^Ly6C^int^ neutrophils were determined by flow cytometry at day 10 post infection. Values are the mean ± SEM for 5–6 mice per group. * = *P* < 0.05.

Further to day 6 of the disease, we investigated the leukocyte infiltration and tissue histology at day 10. While the mock-treated group showed elevated percentages in total CD45^+^ leukocytes and small CD45^+^SSC^lo^ lymphocytes/NKcells, there was no difference in total leukocyte numbers (Figures 6A and B), suggesting MTX-treated mice had an increased number of non-immune cells in infected quadriceps. Moreover, as critical cellular determinants for RRV disease severity, CD11b^+^Gr1^hi^ inflammatory monocytes/macrophages, were comparable between mock and MTX-treated mice in both percentages and numbers (Figure 6C), explaining the equally severe disease state at day 10 (Figure 1A). In histological analysis, there was no difference in immune infiltrates between mock and MTX-treated groups and muscle damage appeared to be comparably severe (Figure 6D). Furthermore, the effect of MTX treatment on the systemic myeloid cell compartment was distinct from day 6. While the cellularities of inflammatory monocytes and neutrophils remained elevated at day 10, the increased percentage of systemic inflammatory monocytes seen at day 6 no longer held in the MTX-treated group (Figure 7), explaining the comparable CD11b^+^Gr1^hi^ myeloid infiltrates and disease scores between two groups.

## Discussion

MTX is a standard treatment for patients who suffer moderate to severe rheumatoid arthritis. As to alphavirus-induced arthritic disease, it is currently used as a treatment option to alleviate chronic symptoms [6], [14]. Here we demonstrate that MTX provides no therapeutic benefit to acute RRVD in mice; rather, it enhances disease onset and severity at the early stage of infection and sustains the peak of the disease symptoms, in comparison to non-MTX-treated mice. The rapid onset of RRVD in MTX-treated mice correlates with a significantly increased influx of inflammatory monocytes into the skeletal muscle tissue, in conjunction with elevated cellularity of systemic CD11b^+^Ly6C^hi^ inflammatory monocytes. These findings raise questions over the therapeutic value of MTX in patients who suffer acute arthritic symptoms during alphaviral infections.

MTX has been initially identified as an anti-metabolite. It has been shown to cause folate depletion and purine inhibition [15], [16]. MTX has been shown to inhibit cell growth and is considered cytotoxic [17]. With such cytoxicity, methotrexate treatment has been reported to associate with undesired side effects and death in mice [18]. Being the first to report on MTX treatment in the mouse model of RRV disease [19], we performed drug titration experiments with 21-day old C57BL/6 mice and determined a most suitable physiological regimen for this study (0.25 mg/kg/day) (data not shown). With this standardized protocol, we did not observe an ameliorating effect of MTX on acute musculoskeletal disease in the context of virus infection. This is in odds with previous studies, which have demonstrated the anti-inflammatory outcome of the drug [20].

Although MTX is commonly used to treat rheumatic disease, its mechanism of action is not clearly defined. MTX is a folate antagonist and its anti-inflammatory effect has been directly linked to inhibition of purine and pyrimidine formation, which results in defective DNA and RNA syntheses [15], [16]. However, the effect of MTX on the immune system is multifaceted, as several effects of MTX cannot be demolished by folic supplementation [21]. MTX suppresses T cell activation and adhesion [22]. MTX treatment can cause increased adenosine release at the inflamed site that dampens leukocyte accumulation [20]. Intriguingly, in our study, MTX enhances leukocyte infiltration at the acute stage of RRV infection, which is concurrent with an early onset of disease symptoms and sustained disease severity. To this end, how do we reconcile these findings? Affirming the diverse effects of MTX on the immune system [21], MTX has previously been shown to act as a strong differentiation factor for immature and undifferentiated monocytic cells *in vitro* [23]. With our treatment regimen, while MTX alone did not trigger monocyte elevation *in vivo*, likely due to the low dosage of MTX administered, in the presence of RRV infection, MTX treatment boosts systemic monocyte generation. This is most likely the result of a synergistic effect arising from proinflammatory cytokines and/or cell differentiation factors produced during acute RRV infection. Such an effect may override immunosuppression reported previously. Interestingly, other myeloid cells, such as neutrophils, were not affected by MTX treatment, indicating that this effect is monocyte/macrophage lineage-specific. Further studies are needed to identify the stimulatory factors involved in this process.

A number of recent studies have established the importance of inflammatory monocyte infiltration in alphaviral disease [8], [12]. In line with these results, we show that early onset disease in MTX-treated mice was accompanied by a significant increase in systemic monocyte cellulartiy and quadriceps infiltration of CD11b^+^ cells. However, little change in NK, T and B cell infiltration was observed between MTX and mock-treated mice at day 6 post infection, highlighting the importance of inflammatory monocytes/macrophages in the immune-pathology of RRVD. Moreover, it has been found that at peak RRVD there is an increase in the number and range of cells found within the inflammatory infiltrate [19]. RRV-infected mice lacking functional T and B cells have been found to display a somewhat reduced clinical score at peak disease, suggesting these cells may contribute to aspects of RRVD [19]. Cells, such as CD4^+^ and CD8^+^ T lymphocytes and dendritic cells may explain the overall percentage increase in inflammatory infiltrates observed at day 10 post infection in mock-treated infected mice. The increased leukocyte percentage in mock-treated infected mice is also likely a result of an increase of non-immune cells in the MTX-treated group. Additionally, low dose MTX has been shown to suppress T cell activation *in vitro* and may therefore contribute to the reduced percentage of CD45^+^ cell infiltrates in MTX-treated RRV-infected mice at peak disease [22].

Virus induced inflammation in joints and skeletal muscle is a distinct characteristic of the RRV mouse model and changes in viral titer can dramatically alter RRVD presentation [19], [24], [25]. Higher viral titers in the serum and also quadriceps of MTX-treated mice at early time points post infection suggest increased viral replication may contribute to the early onset of disease seen in these mice. The evidence for an immunosuppressive effect of low-dose MTX, frequently examined using large cohort studies on associated risk of infection following treatment of RA patients, can often be contradictory [26], [27], [28]. Although, the risk of serious infection in RA patients receiving MTX has been well documented [27], [29], [30]. It is possible that the initial rise in viral titers in MTX-treated mice may be due to an immunosuppressive effect due to MTX treatment, however, the effect of MTX on the immune system in the RRVD mouse model at early times post infection has yet to be examined.

With no licensed vaccine currently available, treatment of RRVD is often symptomatic. Results herein suggest that the use of anti-rheumatic drugs such as MTX should be used with caution when treating early alphaviral-induced arthritis. Although largely successful in the treatment of RA and the chronic rheumatic manifestations occurring in patients previously infected with arthritogenic alphavirus, these results advise against the treatment of acute RRVD, and potentially other viral arthritides, with MTX.
